# Exploring the perspectives of undergraduate allied health sciences, dentistry and nursing students regarding the unpaid clinical placements

**DOI:** 10.1186/s12909-026-09487-y

**Published:** 2026-06-19

**Authors:** Syed Gohar Hussain Shah, Nowshad Asim, Syed Muhammad Junaid, Brekhna Jamil, Abdul Basit

**Affiliations:** 1 Abbottabad International Dental College, AIDC, Abbottabad, Pakistan; 2 Institute of Health Professions Education & Research (IHPE&R), Khyber Medical University, KMU, Peshawar, Pakistan

**Keywords:** Allied health sciences, Clinical education, Dentistry, Nursing, qualitative research, Pakistan, Unpaid placements

## Abstract

**Background:**

Clinical placements are essential for developing competence and professional identity in health professions education. In Pakistan, many undergraduate students in Allied Health Sciences, Dentistry, and Nursing complete unpaid clinical placements, which may impose financial, emotional, and educational challenges. Limited multidisciplinary qualitative research exists to understand these experiences.

**Objective:**

To explore the perspectives of undergraduate allied health sciences, dentistry and nursing students regarding the unpaid clinical placements.

**Methods:**

The qualitative study design involved semi-structured interviews with 22 undergraduate students of Dentistry, Nursing, and Allied Health Sciences programmes in various institutions in Khyber Pakhtunkhwa, Pakistan. Purposive sampling was used to recruit participants. Interviews were held in the Urdu language, taped and transcribed verbatim. Thematic analysis of data was performed in six phases as developed by Braun and Clarke. The use of reflexive note-taking, member checking, and audit trail were strategies used to increase rigor. Informed consent was received and ethical approval granted by the Ethics Review Board KMU-IHPER. The study was carried out in secrecy and anonymity.

**Results:**

Five major themes emerged:

1. Managing Daily Costs and Practical Challenges of Clinical Rotations.

2. Emotional and Psychological Strain During Clinical Placements

3. Gaps in Supervision and Professional Support

4. Challenges Within the Clinical Learning Environment and Workload

5. Shaping Professional Identity and Motivation Through Clinical Exposure

The results were subsequently explained through the Hierarchy of Needs invented by Abraham Maslow, which suggested that the physiological and safety needs that were not met, including financial strain and lack of support, inhibited the capacity of students to participate in higher-order learning, which consequently influenced their well-being and formation of professional identities.

## Background

Clinical education has become an important part of training in the health professions as it allows students to convert theoretical knowledge into practical competence, to develop clinical reasoning, and to internalise professional behaviours. Quality of clinical placements is a crucial determinant of confidence, motivation and readiness to practice independently among learners, and it is commonly described by well-organised supervision, well-articulated learning goals, sufficient resources available, and psychologically safe learning conditions [1]. Nevertheless, the level of realization of these conditions differs significantly between countries, institutions, and fields.

Although central to professional training, clinical placements in most settings are not paid, thus burdening students and adding to the pressures beyond the academic requirements [2]. Although students in high-income environments enjoy a comparatively organised system of clinical education with a consistent level of supervision and formalised feedback mechanisms, they nonetheless report stress-related issues, anxiety, and pressures of work [1, 2] It is therefore likely that in low- and middle-income countries (LMICs), including Pakistan, these pressures are further exacerbated by resource limitations, patient volumes, faculty availability, and inconsistent supervision, further affecting the quality of learning experiences [3].

These challenges can be approached through the Hierarchy of Needs developed by Abraham Maslow. Under this framework, people are less capable of higher-order learning when the lower physiological and safety needs have not been addressed [4]. In the case of unpaid clinical placements, financial stress and insufficient support can restrict students in complete learning, thus impacting their learning prospects and development as professionals [5].

Though there are literature sources that discuss a variety of issues related to clinical education, most of the existing evidence is either field-specific or based on high-income settings. Additionally, there is a dearth of research on unpaid clinical placements in various health fields especially in LMICs like Pakistan. Moreover, most of the current studies are quantitative, and they provide poor understanding of the lived experiences and the mechanisms of action underlying the effects of financial, structural and educational variables on the learning and well-being of students [6, 7].

Students in the Dentistry, Nursing and Allied Health Sciences programmes in Pakistan are particularly lacking in qualitative evidence, although they are heavily engaged in clinical training. Knowledge of the experience of students in these disciplines is crucial to finding out common problems and to discipline-specific variations, and to instructing policy- and context-sensitive changes. A qualitative exploration of these experiences enables a better understanding of the influence of unpaid clinical placements on student engagement, well-being, and professional identity [8].

### Rationale

In Pakistan, undergraduate students in allied health sciences, dentistry and nursing are required to complete unpaid clinical placements, often without financial support from their institutions. This leads to significant financial stress, particularly among students from low-income, rural, or caregiving backgrounds. Existing literature highlights the negative impact of such stress on students’ academic performance, mental health, and career decisions. However, most research focuses on medical students, with little qualitative exploration of the lived experiences of allied health and nursing students [6]. This study aims to fill that critical gap by exploring their perspectives regarding unpaid clinical placements, aligning directly with the objective to understand the academic, emotional, and socioeconomic impact of these placements on students in the Pakistani context [9, 10].

## Methods

### Study setting

The research was carried out in several public and private institutions in Khyber Pakhtunkhwa (KPK), Pakistan, such as Khyber Medical University (KMU) Hazara Campus, Abbottabad International Dental College (AIDC), and Frontier Institute of Medical Sciences (FIMS), and the related clinical training sites.

Clinical placements were done in various healthcare settings such as tertiary care hospitals, teaching hospitals and specialised units, such as Ayub Teaching Hospital, District Headquarters Hospital (DHQ) and AIDC in Abbottabad.

The two settings were chosen because they were viewed as normal clinical training settings in KPK where unpaid placements are common. The use of many institutions allowed to explore the role of different institutional backgrounds, supervision, and resource levels in defining the experiences of students, thus increasing the depth of information and the level of contextualization of its data.

### Study design

The current research applied interpretivist paradigm in a quality exploration research design to study the experiences of the unpaid clinical placements among undergraduate students. Virginia Braun and Victoria Clarke declare that the reflexive thematic analysis was applied to elicit deep information regarding the lived experiences of the interviewees [11].

### Study population

The population of the targeted group was undergraduate students undertaking or recently completed unpaid clinical placements in Dentistry, Nursing and Allied Health Sciences programmes.

The sample consisted of 22 people, which is suitable in representing different disciplines, academic levels, and levels of clinical exposure. Male and female students were chosen to belong to different views. The difference provided an opportunity to speak about similarities and differences in experience on various areas, particularly in terms of workload demands, supervision tendencies, and budget concerns.

### Sampling strategy

It used purposive sampling strategy with the maximum variation strategy that sought respondents that had a immediate experience of unpaid clinical placements. The participants were selected in collaboration with the members of the faculty and clinical supervisors at the KMU Hazara Campus, AIDC, and FIMS and hospitals attached to them.

A preselected group of about 30 eligible students was then outlined on the basis of preset inclusion criteria. Among these 22 students agreed to take part and other students refused to take part owing to lack of time or their lack of interest.

The process of sampling went on until data saturation was reached, which was a goal of collecting data by the researcher to mean that no additional themes or valuable information would be obtained through subsequent interviews.

### Data collection procedure

The data were gathered between six and eight weeks, depending on the clinical rotation of the participants. The interviews were semi-structured and were conducted by using an interview guide, which was developed based on literature and objectives of the study.

The interviews were conducted in Urdu, were typically of the 15–20 min long, and were conducted by the main researcher who had had previously some training in qualitative research and had no immediate evaluative connection with the people being interviewed.

Audio-recorded with permission, all interviews were also complemented by field notes to record the situation and initial impressions.

Informed consent was signed and an information sheet given to the participants before the interview. They were promised to participate willingly, confidentially, and their right to withdraw at any point without any repercussions.

### Data handling

All interviews were recorded and transcribed word-to-word with the consent of participants. Urdu interviews were translated into English and precision was maintained by cross checking transcripts with original transcripts. Transcription involved deleting identifiable data to ensure anonymity of participants. All information was kept in safe and password-protected documents that were accessible only to the researchers. During data analysis and reporting, participants were given their distinct identification codes (e.g., P01-P22) and the study was conducted anonymously.

### Data analysis

The reflexive thematic analysis described by Virginia Braun and Victoria Clarke was used to analyse the data. The analysis was done in six phases:

1. *Familiarisation*: Transcripts were read several times to have a profound knowledge of the data.
2. *Initial Coding*: The dataset was inductively coded into meaningful data segments.
3. *Generating Themes*: Codes were grouped into possible themes which represent patterned answers.
4. *Reviewing Themes*: Themes were narrowed down by comparing them with coded extracts and whole dataset.
5. *Defining and Naming Themes*: Themes were well defined, named, and organized to depict latent patterns and relationships.
6. *Producing the Report*: Themes were confirmed and backed with exemplary participant quotes.

The primary researcher performed coding manually. A second researcher reviewed the selected transcripts and coding decisions in order to increase the rigor of the analysis. Any differences were debated and settled out by mutual agreement. The analysis was intended to place both the similarities in the experiences of the disciplines and differences where they could be realized and also provide a subtle understanding of the data.

### Rigor and trustworthiness

In order to achieve the methodological rigor, the study followed the criteria suggested by Yvonna Lincoln and Egon Guba:

- *Credibility*: This was attained by long interaction with the data, using word-to-letter quotations and member checking, in which participants were allowed to look at summaries of findings and check their accuracy.
- *Dependability*: This is ensured by the use of an audit trail that documents all the methodological and analytical decisions made during the study.
- *Confirmability*: This is supported by reflexive note-taking, which enables the researcher to recognize and critically reflect on the possible biases and assumptions.
- *Transferability*: Strengthened through the inclusion of enriching descriptions of the research situation, respondents and results, allowing readers to determine how it can be applied to other contexts.

Data collection and analysis were carried out simultaneously and saturation of the theme was taken to be reached when no new codes or themes were being added after each consecutive interview was carried out.

## Results

### Theme 1: Managing Daily Costs and Practical Challenges of Clinical Rotations

Students have always spoken about the economic pressure and the cost to bear and the inconveniences and difficulties of practical life. Such difficulties were not limited to independent costs but manifested themselves. to inform students to be able to engage in clinical learning in a constant way.

### Struggling with transport and daily travel costs

One of the main and repeated financial burdens was revealed to be transportation that had a great impact. The involvement of students in clinical placements on a daily basis.

#### Participant quotes

P03: “A huge portion of my monthly bills is just spent on transport. Because we are not paid, it is quite difficult to get to from day to day.”

P08: “Some clinical sites are very distant. We work early and go home and drive the day and the price is only keeps adding up.”

P21: “I have to ask my parents to give me more money to travel every week and I feel guilty because they do it. are already managing so much.”

This implies that affordability is not the only financial pressure that affects it. consistency of attendance and emotional liability, which could minimize student attendance. participation in clinical learning exercises.

### Absorbing hidden costs of clinical requirements

Besides transport, students claimed that there are several expense areas that are involved with clinical outside the plane of charge. participation, such as uniforms and equipment and daily necessities.

#### Participant quotes

P11: “We purchase our uniforms and decent shoes and all of this is costly. No one puts a pin, though we must have them on every day.”

P06: “I would not purchase food during the placement hours at times due to the need to save money later in the day.”

P15: “Skills practicing equipment is expensive. We purchase all by ourselves and it is a burden after some time.”

These results show that unpaid placements make students financially liable, making them compromise the basics and the educational participation, which may also further undermine their health and education.

### Navigating unpredictable schedules and rotation changes

Students mentioned that frequent rotation shifts which were not communicated often resulted in more. logistical and financial stress.

#### Participant quotes

P02: “Every time the rotation is different, it gets different, the handling, the costs, the time. It is a terribly stressful kind of planning.”

P07: “I would arrive at one ward and they would tell me that they had changed something somewhere. else. It consumes time and money and we are powerless.”

P10: “Because the rotations are rotating and I am not getting due notice, at times I. am using alternative routes or using a number of vehicles. It becomes exhausting.”

P18: “When we do not know what time we shall have so precisely it makes a difference of it all, attending, travelling or even our trust. We are always guessing.”

This echoes the fact that structural discrepancies in clinical scheduling intensify financial as well as. logistical issues, which add to uncertainty and low efficiency among students. involvement in clinical activities.

### Theme 2: emotional and psychological strain during clinical placements

It was noted that students had severe emotional and psychological problems during unpaid clinical. placements. High requirements in the workplace, fear of being, were the sources of these pressures. poor performance and the ongoing stress between the academic and clinical workload.

### Experiencing stress and mental fatigue

The cumulative demands of that long time in the clinical were often overwhelming to many students. academic requirements, and they are in constant pressure to prove their competence.

#### Participant quote

P12: “The mental strain builds up. I simply lack any energy on some days. after placement.”

P05: “Every day feels heavy. Work like full-time employees and go home exhausted.”

P09: “Balancing the classes and clinical is stressful. There’s no time to rest well.”

This implies that prolonged workload requirements cause exhaustion which could lower cognitive learning competence and restrict student capability to reflect on their practice.

### Anxiety related to clinical expectations and environment

Students reported a constant anxious feeling around clinical expectations.

#### Participant quote

P01: “Before reaching hospital I am nervous thinking of what job have been assigned. to us that day.”

P14: “I am never sure whether I am doing something right. There is always this trepidation of correction before all.”

P22: “Sometimes, it is overwhelming at the environment. I panic in a bid to keep abreast of it. Whether I am asking what is going on around me.”

This implies that pressurized situations and fear of judgment can be a barrier to confidence. and impact negatively active involvement in clinical tasks.

### Feeling unsupported emotionally or institutionally

Students indicated that although they might experience stress, anxiety, or discouragement, there were none. institutional means of psychological support.

#### Participant quote

P16: “No actual room to discuss the way we are feeling. We simply do it even when we are stressed. keep going.”

P11: “Sometimes it feels like it is nobody, it is like they do not notice how we are under emotional pressure.”

P20: “I support each other, we are students, and, at the other end, there is nothing, the institution. so that we can cope with the pressure.”

This indicates the inability to support the institutions making it more stressful. be a source of emotional exhaustion.

### Theme 3: gaps in supervision and professional support

Students kept stressing on the role of supervision in their clinical learning. experiences. But there were perceived to be gulfing holes in the provision, accessibility, and intervention of supervisors in various departments and hospitals.

### Limited availability and accessibility of supervisors

Lack of supervision or receiving any guidance in clinical rotations in time was reported by many students. because of the excessive workload of their supervisors, which result in their reduced contact with students.

#### Participant quote

P03: “We are not sure most of the times with whom to report. We are too busy, you are too busy, supervisors. left to work things out ourselves.”

P08: “There are times that we spend a half of the shift in search of the one in charge due to the fact that we are not. clearly told.”

P19: “Lack of proper supervision. Here we are, and we do not receive much guidance. unless we have to push for it.”

This implies that the lack of supervision minimizes the chances of adviced learning and departure. students searching clinical tasks on their own.

### Inconsistent or insufficient feedback

Respondents said that when the feedback is provided, it was typically concise, inconsistent or directed. corrective remarks as opposed to constructive advice.

#### Participant quote

P12 “When we commit mistakes, we are given the instruction that we should not be repeated and never is it told us what the right is. method is.”

P07: “We do not receive proper feedback that often. And the rest of the times it is a hasty correction or self-explanatory instructional intervention.”

P22: “I do not wish to be taught how to do it next time, I require feedback which would help me to get. better.”

This implies that the skills can be capped due to the lack of formal feedback and impairment. learning progression.

### Needing more guided in skill development and confidence

In the case where there was no procured support, students reported that they felt unsure, unprepared and unwilling to conduct. procedures on their own.

#### Participant quote

P05: “From time to time we are called in to assist with procedures that we have never practiced. It is scary when there is no one to guide.”

P10: “We want to learn, but nobody demonstrates to us how to do it step-by-step.” “It affects our confidence in the sense that we are expected to do things without have proper guidance.”

P13: “If a person teaches us properly we can perform well. But without such guidance, everything is confusing.”

This reflects how lack of mentorship can reduce confidence and restrict development of clinical competence.

### Theme 4 challenges within the clinical learning environment and workload

Students described several challenges in the clinical learning environment affecting their ability to effectively learn and confidently engage in patient care.

### Managing heavier clinical workload

Participants said that they were given responsibilities that were far exceeding the training level.

#### Participant quote

P16: “Because there’s not a lot of staff, they give us more work that we can do. It gets stressful and it affects our daily learning.”

P09: “I am just doing the same old stuff all the time. “It gets boring and we don’t learn much with it.”

P22: “When the workload was too great. We were asked to take care of patients are just ordinary personnel despite the fact that we are students.”

This implies that overworking students can leave them in the service positions, depriving them of meaningful roles.

### Limited opportunities of structured learning

Many descriptions of passive, unstructured environments of learning existed. or completely relying on individual initiative.

#### Participant quote

P21: “Nobody told proper procedures in the lab. They were only instructed to watch. sometimes not even that.”

P03: “No organised instruction. We tend to observe or attempt to learn things by their own.”

P19: “In a teaching setting, there would be a lot more learning. But most days we’re not guided through the skills.”

This is an indication of the inexistence of organised teaching structures, which decreases the efficacy of clinical training.

### Unequal or unclear allocation of clinical tasks

In some way or another, students highlighted discrepancies in how activities were allocated. there were those overloaded and some underserved.

#### Participant quote

P14: “There is certain students who have a better chance and some students are not taken notice of. It depends on who the supervisor prefers or whom he or she believes can work quick. It doesn’t feel fair.”

This implies there is a possibility of inequities in exposure to learning, which can impact on competency. development.

### Theme 5: shaping professional identity and motivation through clinical exposure

Students reported all the time how the unpaid clinical placements affected their professional identity, their willingness to make a career in the health sector and their concept of themselves in their future professions.

### Building or struggling with professional confidence

Many participants in the interview said that clinical exposure helped them build confidence or, in some cases, brought out gaps that left them feeling insecure.

#### Participant quote

P05: “When I do something right, I feel more confident. But without guidance, sometimes I am a lot of doubting.”

P21: “Lab Samples, Actual Handling, " I feel that when I was handling actual lab samples, I had confidence, but when no one was correcting us I didn’t know if I was doing things right or not.

P12: “Some days, I feel like I’m on the right track to becoming a professional and other days, I feel totally lost.”

This implies that the availability of feedback and is closely associated with development of confidence.

### Uncertainty about role clarity and purpose

A number of students expressed concern regarding their professional boundaries, roles and responsibilities.

#### Participant quote

P22: “I was at times not aware of what job would be. People were not telling anybody, we were doing. you what their relation is with becoming a technologist.”

P17: “This is because there is no outright guidance on what to or what not to do by the nursing students. The reason is, We simply follow whoever will be there.”

This reflects gaps in role clarity, which may hinder professional identity formation.

### Influence of clinical experiences on future career aspirations

Positive interactions led to an increase in motivation and negative interactions caused some to question whether the profession was right for them.

#### Participant quote

P08: “Some days the experience motivates me to stay in this career and there are some days when I consider that I might have picked the wrong field.”

P02: “If there was proper training and support I would feel better about my future.” Right now, I feel unsure.”

This indicates that clinical experiences play a critical role in shaping long-term career commitment.

## Discussion

This paper analysed the experiences of Allied Health Sciences, Dentistry, and Nursing undergraduate students on unpaid clinical placements in the Pakistani province of Khyber Pakhtunkhwa and the interaction of financial, emotional, supervisory, and environmental constructs in learning and professional development. The results suggest that unpaid clinical placements do not only influence both access to clinical learning and ultimately student motivation, well-being and future career intentions. This result aligns with the international evidence indicating that unpaid placements cause financial inequalities and have adverse impacts on the engagement of students in health professions education [12, 13].

Financial load proved to be one of the most important structural limitations affecting the involvement of students in clinical training. Students complained about the repetitive out of pocket costs in the form of transport, food, uniform and equipment that went beyond the inconvenience to a level of consistently restricting access. This implies that financial precarity affects how students attend school on a regular basis, focus on placements and engage in learning in all its activities. In line with other studies conducted internationally, it was found that hidden costs, in a disproportionate manner, affected students with lower socioeconomic status and contributed to inequalities in accessing training opportunities [13, 14]. In the given study, financial insecurity emerged as the primary factor reducing student involvement in clinical learning, which supports structural inequities in educational access in health professions. Economic pressures had a strong association with emotional and psychological stress. Students have reported fatigue and anxiety, the fear of mistakes, and emotional exhaustion, especially in high-demand and resource-constrained clinical settings. The findings are consistent with the world-wide literature that recognizes clinical placements as a primary source of stress among health professions students in low-supervision and low-support context, with emotions having a detrimental impact on motivation and ability to gain full benefit by use of clinical exposure [15].

Supervision and feedback proved to be very important in the learning process of students. The participants complained of patchy supervision, vague expectation, and little constructive feedback, which made them unsure of how they were developing skills and forming their identity professionally. The existing studies indicate that in health professions education, good supervision is closely linked to better learning experience, confidence, and professional socialization, but in resource-limited environments, clinical settings compel supervisors to be peripheral, and students become peripheral learners, which are part of an informal learning experience that is part of a hidden curriculum.

The experience of the students was also affected by the clinical learning environment. High population wards, workloads and service priorities prevented the possibility of organized learning and reflective practice. Other contexts have also reported similar struggles, with disorderly clinical settings limiting meaningful interactions with patients, and a significant number of students feeling frustrated by failures of consistent instruction and absence of explicit learning goals [15, 16]. Although a number of students have mentioned gaining confidence in interacting with patients, many were frustrated by poor organization, the lack of consistent teaching, and a deficiency of clear learning outcomes. This implies that clinical training might not be effective because the anticipations of the curriculum do not reflect clinical realities.

The results of the research would be decipherable through the Hierarchy of Needs by Maslow, which will be a useful theory to support the research on the effects of unmet basic needs on learning. In unpaid clinical placements, financial hardship, neglect, and the high-stress conditions seem to be a barrier to higher-order learning processes and career growth, whether students are motivated or committed to them or not [4, 14, 17] This underscores the role of addressing underlying requirements in order to have successful clinical education.

Altogether, this research adds multidisciplinary qualitative research to the body of literature on health professions education in Pakistan. The analysis of unpaid clinical placements in Allied Health Sciences, Dentistry, and Nursing programmes reveals that financial issues, lack of supervision, and clinical setting conditions interact to influence the experiences of students. These lessons inform the importance of institutional and policy-level changes, such as the enhancement of the supervision framework, the enhancement of the capacity to align educational institutions and clinical placements, and the acknowledgment of the financial strain on students. These are the issues that must be addressed to bring equity, effective, and sustainable clinical education to Pakistan.

### Limitations of the study

There are a number of limitations that are associated with this study and which should be taken into consideration when interpreting the results. To begin with, the sample was relatively small and did not exceed 22 participants, which is acceptable in the context of qualitative research that strives to achieve depth, but it might be a problem in terms of transferability of results to other contexts. Second, the sample was selected based on a small population of institutions in Khyber Pakhtunkhwa and thus the results are context-specific such that they may not be representative of other parts of Pakistan. Third, the sample also covered students across several fields such as Dentistry, Nursing, and Allied Health Sciences. Although this method made it possible to discover common trends, it might not have developed the discipline-specific differences in an equal measure. These variations can be investigated further by discipline-based or comparative designs in future studies. Fourth, the data were self-reported and therefore vulnerable to recall bias and social desirability bias. Even though the participants were assured of confidentiality and asked to be truthful, they may have minimized or overemphasized some of their experiences. Lastly, the qualitative data interpretation is, by definition, controlled by the outline of the researcher despite the strategies used to promote trustworthiness, such as taking reflexive notes and having an audit trail, as the interpretation of the latter cannot be objective. The reduction of bias was achieved by a close analysis and reporting transparency, but one could not presume that complete objectivity is achieved.

## Conclusion

Undergraduate health professions students in Khyber Pakhtunkhwa are experiencing a lot of financial, psychological, and educational burden through unpaid clinical placements. Those challenges affect the engagement of students in the learning process, their well-being, and the formation of professional identity. To achieve fair and efficient clinical learning in this regard, financial limitations, enhanced supervision, and better organization of clinical learning settings are fundamental issues that should be addressed.

## Data Availability

The datasets generated and/or analyzed during the current study are not publicly available due to ethical considerations and protection of participant confidentiality but are available from the corresponding author on reasonable request.

## Abbreviations

AHS: Allied Health Sciences
AIDC: Abbottabad International Dental College
ASRB: Advanced Studies and Research Board
BDS: Bachelor of Dental Surgery
BS: Bachelor of Science
BSN: Bachelor of Science in Nursing
KMU: Khyber Medical University
LMICs: Low- and Middle-Income Countries
MHPE: Master of Health Professions Education
PNC: Pakistan Nursing Council
SDT: Self-Determination Theory
FIMS: Frontier Institute of Medical Sciences
SOP: Standard Operating Procedure
WHO: World Health Organization
UCP: Unpaid Clinical Placement
